# Plagiochiline A Inhibits Cytokinetic Abscission and Induces Cell Death

**DOI:** 10.3390/molecules23061418

**Published:** 2018-06-12

**Authors:** Nicole S. Stivers, Ashraful Islam, Elsa M. Reyes-Reyes, Lavona K. Casson, José C. Aponte, Abraham J. Vaisberg, Gerald B. Hammond, Paula J. Bates

**Affiliations:** 1 Department of Biology, University of Louisville, Louisville, KY 40208, USA; nsstiv01@gmail.com; 2 Department of Medicine, University of Louisville, Louisville, KY 40202, USA; ashrafulislam01@gmail.com (A.I.); emreye01@email.arizona.edu (E.M.R.-R.); lavona.casson@louisville.edu (L.K.C.); 3 Department of Clinical Biochemistry, University of Tabuk, Tabuk 71491, Saudi Arabia; 4 Division of Pulmonary, Allergy, Critical Care, and Sleep Medicine, University of Arizona College of Medicine, Tucson, AZ 85724, USA; 5 Department of Chemistry, University of Louisville, Louisville, KY 40292, USA; jose.c.aponte@nasa.gov (J.C.A.); gb.hammond@louisville.edu (G.B.H.); 6 Departamento de Ciencias Celulares y Moleculares y Laboratorios de Investigacion y Desarrollo, Facultad de Ciencias y Filosofía, Universidad Peruana Cayetano Heredia, Lima 15102, Peru; abraham.vaisberg@upch.pe

**Keywords:** plagiochiline A, *Plagiochila disticha*, cytokinesis, abscission

## Abstract

We previously reported on the isolation and biological activities of plagiochiline A (**1**), a 2,3-secoaromadendrane-type sesquiterpenoid from the Peruvian medicinal plant, *Plagiochila disticha*. This compound was found to have antiproliferative effects on a variety of solid tumor cell lines, as well as several leukemia cell lines. Other researchers have also noted the cytotoxicity of plagiochiline A (isolated from different plant species), but there are no prior reports regarding the mechanism for this bioactivity. Here, we have evaluated the effects of plagiochiline A on cell cycle progression in DU145 prostate cancer cells. A cell cycle analysis indicated that plagiochiline A caused a significant increase in the percentage of cells in the G_2_/M phase when compared with control cells. When cells were stained and observed by fluorescence microscopy to examine progress through the mitotic phase, we found a significant increase in the proportion of cells with features of late cytokinesis (cells connected by intercellular bridges) in the plagiochiline A-treated samples. These results suggest that plagiochiline A inhibits cell division by preventing completion of cytokinesis, particularly at the final abscission stage. We also determined that plagiochiline A reduces DU145 cell survival in clonogenic assays and that it induces substantial cell death in these cells.

## 1. Introduction

In the early 1990s, an ethnobotanical project was initiated to investigate the chemical and biological properties of the medicinal plants that had been used by the Aguaruna tribe from the Peruvian rainforest. This project was supported by an International Cooperative Biodiversity Grant (ICBG) and because the plants chosen for the study had been prescreened for human use by the Aguaruna people, they provided higher frequencies of bioactive secondary metabolites than the flora as a whole [1].

*Plagiochila disticha (Plagiochilaceae)*, a liverwort (bryophyte), was among the species chosen for the study, and we previously reported on components that displayed antiproliferative activity against cancer cells, isolated by bioactivity-guided fractionation [2]. One of the components identified in that study was plagiochiline A (**1**, see Supplementary Figure S1), a 2,3-*seco*-aromadendrane-type sesquiterpenoid that had been previously identified in other species and reported to display insect antifeedant activity against the African armyworm, *Spodoptera exempta* [3], as well as cytotoxic effects on murine leukemia cells [4]. We found that plagiochiline A displayed significant antiproliferative activity against a variety of solid tumor cell lines, including those derived from prostate, colon, breast, lung, and cervical cancers, as well as several leukemia cell lines [2]. Antiprotozoal activity was also observed [2]. Several other plagiochiline derivatives have been reported (plagiochilines B–X), and many of them have shown different biological activities [5]. For instance, plagiochiline C can significantly inhibit platelet aggregation, whereas plagiochiline M produces abdomen and wing malformations in adult Fall armyworms, *Spodoptera frugiperda*, thereby preventing mating [5,6,7].

The goal of the current study was to investigate how plagiochiline A inhibits the proliferation of cancer cells. Because many plant-derived anticancer compounds cause cell arrest during mitosis [8], we decided to first determine the effect of plagiochiline A on the distribution of cells in each phase of the cell cycle. DU145 cells, which are derived from a metastatic hormone-independent prostate cancer, were used throughout our study.

## 2. Results

**Figure 1:**
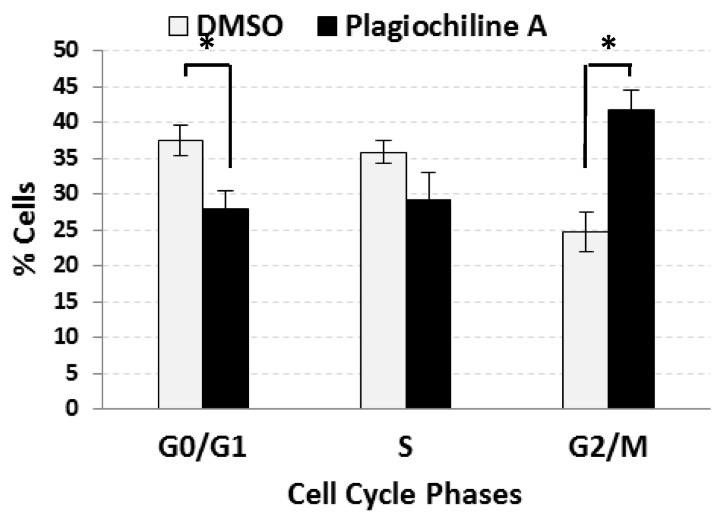
Plagiochiline A induces G_2_/M cell cycle arrest in DU145 cells. DU145 prostate cancer cells were treated for 24 h with 5 µM plagiochiline A or vehicle control (DMSO) and processed for cell cycle analysis by flow cytometry. Columns represent the mean of three independent experiments with bars representing standard error. Comparing plagiochiline A-treated vs. vehicle control cells, the increase in G_2_/M phase and the decrease in G_0_/G_1_ phase were statistically significant (*, *P* = 0.014 and 0.045, respectively).

DU145 cells were incubated for 24 h in the presence of 5 µM (1.75 µg/mL) plagiochiline A or an equivalent amount of vehicle (DMSO). To determine the percentage of cells in various phases of the cell cycle, a flow cytometry analysis of cells stained with propidium iodide (to measure DNA content) was used. The results revealed a statistically significant increase in the percentage of cells present during the G_2_/M phase for plagiochiline A-treated cells when compared with control cells (41.7% compared with 24.8%, *P* = 0.014), with corresponding decreases in the percentages of G_0_/G_1_ and S phase cells, as shown in Figure 1 (see also Supplementary Figure S2). There was no significant difference between the control treatment (DMSO) and untreated cells (Supplementary Figure S2). These data indicate that plagiochiline A causes an arrest of the cell cycle during the G_2_/M phase.

**Figure 2:**
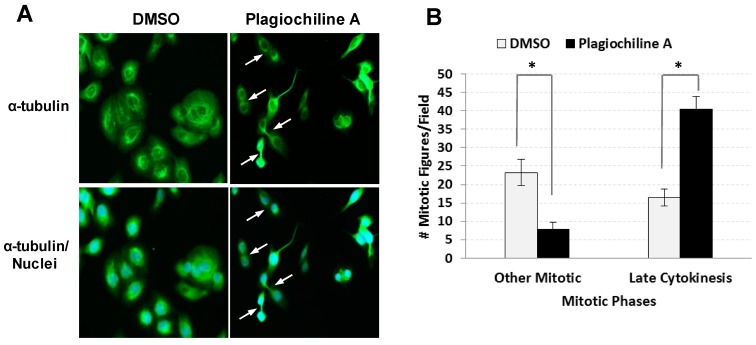
Fluorescence microscopy indicates defective cytokinesis in DU145 cells treated with plagiochiline A. DU145 cells were plated on glass cover slips and incubated 24 h at 37 °C. Cells were then treated for 48 h with 5 µM plagiochiline A or vehicle control (DMSO). Cells were washed with PBS, fixed with 4% paraformaldehyde, and permeabilized with 1% Triton X-100. Nuclei were stained with 4′6-diamidino-2-phenylindole (DAPI, blue) and α-tubulin was stained using anti-α-tubulin antibody labeled with fluorescein isothiocyanate (FITC, green). (**A**) Representative photomicrographs with arrows indicating cells arrested at late cytokinesis (i.e., nascent daughters remain attached by intercellular bridges). (**B**) Graph showing the number of mitotic figures observed per field examined (500 cells). Columns represent the mean of four independent experiments with bars representing standard error. Comparing plagiochiline A-treated vs. vehicle control cells, the increase in late cytokinesis and the decrease in other mitotic figures were statistically significant (*, *P* = 0.001 and 0.0084, respectively).

The G_2_/M phase of the cell cycle consists of the following: (i) the G_2_ “gap”, when proteins are synthesized and cell size increases; and (ii) the mitotic phase (M), where both mitosis (nuclear division) and cytokinesis (cytoplasmic division) occur. To determine if plagiochiline A-treated cells were arrested at a specific stage of the G_2_/M phase, we examined cells that had been stained with an anti-tubulin antibody (to visualize microtubules) and 4′,6-diamidino-2-phenylindole (DAPI, which binds DNA and mrks the chromosomes), allowing us to identify cells at various stages of the mitotic phase based on their appearance and microtubule localization. Using this approach, we examined DU145 prostate cancer cells after incubating them for 48 h with 5 µM plagiochiline A or with an equivalent amount of vehicle (DMSO) as control. We noticed that in the plagiochiline A-treated sample, when compared with controls, there were more cells that appeared to have completed mitosis, but were still connected by intercellular bridges (Figure 2A and Supplementary Figure S3), corresponding to “late cytokinesis”. Quantitation of cells in various stages of mitosis confirmed that there was a statistically significant (*P* = 0.001) increase in cells at this late cytokinesis stage following 48 h of plagiochiline A treatment (Figure 2B). Apart from this overrepresentation of “late cytokinesis” cells, the morphologies of mitotic cells in the plagiochiline A-treated sample were similar to controls (Supplementary Figure S4).

**Figure 3:**
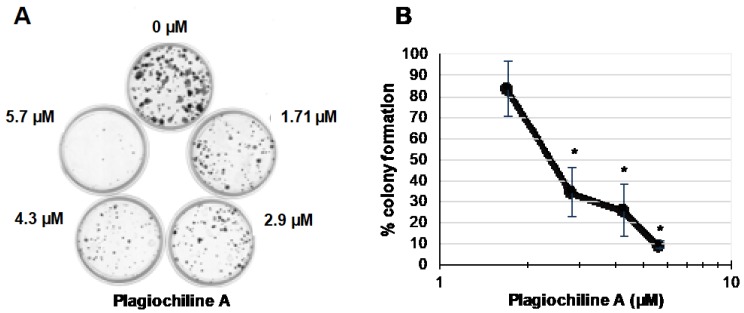
Plagiochiline A inhibits DU145 prostate cancer cell survival in clonogenic assays. DU145 cells were plated at low density in 60 mm plates and incubated in the presence of various concentrations of plagiochiline A, as indicated in the figure. After 15 days of incubation, cells were washed with cold phosphate-buffered saline (PBS), fixed with paraformaldehyde, and stained with crystal violet solution. (**A**) A photograph of one representative experiment is shown; (**B**) Graph showing number of colonies observed in plagiochiline A-treated samples as a % of colony number in control samples (treated with equivalent amount of DMSO vehicle). Data points represent the mean of three independent experiments ± standard error. Values indicated by * were significantly reduced compared to control cells without plagiochiline A (*P* ≤ 0.006).

To further evaluate the potential anticancer efficacy of plagiochiline A, a clonogenic assay was performed to test the effect on DU145 cell survival. DU145 cells were plated sparsely in 60 mm plates (300 cells per plate) and were treated on the following day to give various final concentrations of plagiochiline A: 0 µM (untreated), 1.7 µM (0.6 µg/mL), 2.9 µM (1.0 µg/mL), 4.3 µM (1.5 µg/mL), and 5.7 µM (2.0 µg/mL). Control samples received an equivalent amount of vehicle solution. After 15 days, the number of colonies was counted and results from three independent experiments revealed that plagiochiline A significantly decreased DU145 cell survival at concentrations of 2.85 µM and higher, as illustrated in Figure 3. Plagiochiline A treatment reduced both the number and size of colonies formed (Figure 3). We also determined that the effect of plagiochiline A on DU145 cells was cytotoxic, rather than only cytostatic; flow cytometry analysis showed that a substantial proportion of cells became positive for annexin V and/or propidium iodide staining following treatment with plagiochiline A (Supplementary Figure S5).

Taken together, our data indicate that plagiochiline A induces G2/M cell cycle arrest as a result of failure to complete cytokinesis, which likely contributes to its cytotoxic effect on cancer cells.

## 3. Discussion

Cytokinesis is a complex process that occurs in parallel with mitosis during the M phase of the cell cycle, and which results in the physical conversion of one cell with two nuclei into two mononuclear cells. Beginning during the anaphase portion of mitosis when chromosomes begin to segregate, and completing after telophase when two daughter nuclei are fully formed, the process involves coordinated actions of the cytoskeleton, vesicle trafficking systems, and the cell cycle engine [9]. At the beginning of cytokinesis, the mitotic spindle is involved in specifying the cleavage plane. The microtubules of the mitotic spindle reorganize to form the central spindle, which is a dense bundle of non-kinetochore microtubules. Signaling between this spindle midzone and the cell cortex specifies where the actomyosin ring assembles [10]. Subsequently, actin and myosin act together to form a contractile ring that “tightens” around the middle of the cell to create a cleavage furrow and then begin partitioning the cytoplasm into two. During telophase, the cleavage furrow ingresses further to give rise to two nascent daughter cells that remain connected to each other by a narrow intercellular bridge containing a microtubule-rich midbody. In the final stages of cytokinesis, the midbody is severed through a process termed “abscission”, driven by extensive membrane trafficking and cytoskeletal remodeling [11,12,13].

Our finding that plagiochiline A induces an accumulation of cells connected by intercellular bridges indicates that this compound blocks cytokinesis, specifically at the abscission stage. Abscission occurs by the sequential assembly of the endosomal sorting complexes required for transport (ESCRT) machinery, which progressively constricts the plasma membrane until its cleavage. Among the four multi-subunit protein complexes (ESCRT 0-I-II-III) that are capable of sculpting biological membranes, extensive studies have shown the fundamental role of ESCRT-III in abscission. The ESCRT-III complex in mammalian species comprises a group of proteins, known as charged multivesicular body proteins/chromatin remodeling proteins (CHMPs), and the increased sodium tolerance-1 (IST1) homolog. Recruitment of ESCRT-III at the midbody is thought to mediate the final membrane separation by coordinating membrane and cytoskeletal remodeling events [13]. A specialized midbody protein, centrosomal protein of 55 kDa (CEP55), makes direct interactions with TSG101 and ALIX to facilitate recruitment of ESCRT-III. Additional proteins, such as microtubule interacting and trafficking domain containing 1 (MITD1) and spastin, interact directly with ESCRT-III subunits to provide key functions, including membrane tethering and microtubule clearance [13].

Abscission is tightly regulated to avoid cell cycle progression in cells that have undergone incomplete or faulty division. The abscission checkpoint, also called “NoCut”, is activated in cells with chromosome segregation defects, and depends on Aurora Kinase B-mediated phosphorylation of CHMP4C to delay abscission. Several regulators of this checkpoint have been reported. Thoresen et al. describe ANCHR (Abscission/NoCut Checkpoint Regulator; ZFYVE19), which functions through binding to the ATPase VPS4, a component of the ESCRT machinery. When there are DNA segregation defects, ANCHR associates with VPS4 at the midbody ring in concert with CHMP4C. This association prevents VPS4 relocalization to the abscission zone, thereby blocking abscission [14]. Cellular stresses, such as DNA replication stress, chromatin bridges, high tension in the intercellular bridge (ICB), or defects in nuclear pore complex assembly (NPC), can also induce abscission checkpoint arrest, but it remains unknown how cytokinetic stresses are sensed and relayed to Aurora B activity [15].

Actin cytoskeleton dynamics also play an important role throughout cytokinesis, including in actomyosin ring assembly, contraction of the actomyosin ring, and formation of the intercellular bridge [16]. Inhibition of the actin cytoskeleton dynamics by actin inhibitors or knockdown of actin regulators has been reported to cause a delay of the M phase of the cell cycle, and to inhibit cytokinesis stage progression. Although plagiochiline A also causes an arrest of the cell cycle at the G_2_/M phase, the morphology of cells treated with plagiochiline A is inconsistent with targeting major cytoskeletal proteins (actin, myosin, etc.). Inhibition of the actin cytoskeleton dynamics has been reported to affect multiple phases of mitosis/cytokinesis and causes a cell morphology characterized by an increase in the number of multinucleated cells, chromatin condensation, and mitotic spindles [17,18,19]. By contrast, our observations suggest that plagiochiline A specifically inhibits abscission without affecting other mitotic processes.

In summary, our results suggest that the mechanism of plagiochiline A is distinct from other cytotoxic plant-derived compounds that cause cell cycle arrest in the mitotic phase, including numerous agents that have proven clinically effective in treating cancer [20]. For example, the vinca alkaloids, vinblastine and vincristine, bind with high affinity to the ends of microtubules, which inhibits the movement of chromosomes to the spindle equator during prometaphase and blocks the metaphase–anaphase transition [21]. Paclitaxel, docetaxel, and ixabepilone block mitosis by stabilizing tubulin polymers (which form the microtubules of the spindle), and thereby inhibit the disassembly of microtubules [8,22].

Although there are many anti-mitotic compounds currently used as anticancer agents [8], there are only a few small molecules in development that specifically target cytokinesis, and even fewer that can specifically block at the abscission stage [23,24]. However, there is substantial interest in developing novel inhibitors of cytokinesis as anticancer agents, and small molecules targeting several proteins that are involved in coordinating the early part of cytokinesis (e.g., polo-like kinases, Aurora kinases, and kinesins) have already shown promise in preclinical and clinical studies. Targeting abscission has not been as widely pursued, most likely because it is less well-understood and very few specific inhibitors are available. Only a few compounds have been previously reported to specifically induce cytokinesis failure by inhibiting the abscission step, including synthetic dynamin inhibitors [25,26] and TOPK inhibitors [24]. To our knowledge, plagiochiline A is the first reported plant-derived compound to inhibit completion of cytokinesis by specifically blocking abscission.

Identifying the molecular mechanisms that mediate abscission failure in plagiochiline A-treated cells will require further research, but such studies could provide important information. Induction of late cytokinesis failure is essential in a number of physiological processes, such as sperm meiosis and megakarypoiesis [27,28]. Abscission failure can also lead to aneuploidy and may be a contributing factor in the development of cancer [29], so it is unsurprising that it has been linked to known carcinogens [30,31]. Conversely, there is emerging evidence that targeting cytokinesis is a valuable anticancer approach [23,24]. As summarized above and in several additional reviews [32,33,34,35], our knowledge of the abscission stage of cytokinesis has increased dramatically in recent years, with discoveries such as the role of ESCRT multi-protein complexes and the abscission checkpoint. Plagiochiline A is chemically distinct from other small molecules that inhibit cytokinesis [23,24,25,26,36,37,38,39,40,41,42,43,44,45] and appears to have a unique mechanism of action that specifically targets abscission. Therefore, future studies of plagiochiline A have the potential to reveal additional new insights into the process of cytokinesis and to lead to novel anticancer strategies.

## 4. Materials and Methods

### 4.1. Plagiochiline A

Plagiochiline A was isolated from *Plagiochilia disticha (Plagiochilaceae)*, as described in [2]. For treatment of cells, plagiochiline A was solubilized in DMSO at a concentration of 6 mg/mL and diluted to the desired concentration with either sterile water or cell culture medium. Control treatments used vehicle solutions containing an equivalent amount of DMSO.

### 4.2. Cell Culture

DU145 cells (hormone-independent prostate carcinoma) were obtained from American Type Culture Collection (ATCC) and routinely incubated in a humidified incubator at 37 °C with 5% CO_2_. The cells were grown in DMEM (Dulbecco’s Modification of Eagle’s Medium, from HyClone (Logan, UT, USA), supplemented with 10% heat-inactivated fetal bovine serum (FBS), 62.5 µg/mL penicillin, and 100 µg/mL streptomycin (Hyclone Laboratories, Logan, UT, USA).

### 4.3. Flow Cytometry

DU145 cells (8 × 10^5^) in supplemented medium were plated into flasks (25 cm^2^) for 24 h. After incubation, the cells were treated for 24 h by addition of plagiochiline A or vehicle (containing equivalent DMSO) directly to the culture medium to give the final concentration indicated in the figures or legends. After treatment, the cells were harvested by trypsinization. For cell cycle distribution, the cells were fixed, and stained with propidium iodide using the Cycle Test Plus kit (Becton Dickinson, Franklin Lakes, NJ, USA). Three independent experiments were performed, and *p*-values to assess differences between treatments were calculated using Student’s *t*-test. For cell death detection, the cells were stained with Annexin-V-FITC and propidium iodide using the Apoptosis Detection Kit (BD Biosciences, Mountain View, CA, USA), according to the manufacturer’s instructions. The cells were then analyzed by flow cytometry using a FACScalibur cytometer (BD Biosciences, Mountain View, CA, USA) and FlowJo program (Tree Star, Inc., Ashland, OR, USA).

### 4.4. Immunofluorescence

DU145 cells (1 × 10^5^) in complete culture medium were plated into each well of a 6-well plate containing 18 mm diameter glass cover slips for 24 h. The cells were untreated or treated with 5 µM plagiochiline A or an equivalent volume of vehicle (DMSO) and incubated for 48 h. After incubation, the cells were washed three times with PBS (phosphate-buffered saline), fixed in 4% paraformaldehyde in PBS for 30 min at room temperature, and washed three times for five minutes with PBS. The cells were permeabilized with 1% Triton X-100 in PBS containing 0.02% BSA (bovine serum albumin). Nonspecific binding sites were blocked for 60 min with 2% BSA in PBS. The fixed cells were incubated for 60 min at 37 °C with anti-α-tubulin monoclonal antibody (mouse IgG_1_ isotype) conjugated to fluorescein isothiocyanate, isomer I (FITC) from Sigma (dilution 1:100). After washing, the cover slips were mounted on glass slides with SlowFade^®^ Gold Antifade (Molecular Probes, Eugene, OR, USA). Immunofluorescence was documented with an Olympus Fluoview FV500 Laser Scanning Confocal Microscope (Center Valley, PA, USA). To determine the proportion of cells in various stages of the mitotic phase, fields of 500 cells were examined and mitotic figures were counted and assigned to late cytokinesis or earlier stages of mitosis based on the pattern of fluorescent staining. Four independent experiments were performed and *P*-values to assess differences between treatments were calculated using Student’s *t*-test.

### 4.5. Clonogenic Survival Assay

Cells (3 × 10^2^) in fresh complete medium were plated into 60 mm culture dishes for 24 h. After incubation, the cells were untreated or treated with different concentration of plagiochiline A or an equivalent volume of vehicle (DMSO). The cells were allowed to grow until visible colonies formed (15 days). Cell colonies were fixed with 4% paraformaldehyde in PBS and washed in PBS. The cells were stained with 0.25% crystal violet in 25% methanol, washed and air-dried, and then counted. Three independent assays were performed.

## Supplementary Materials

The following are available online: Supplementary Figures file containing Supplementary Figure S1: Structure of plagiochiline A; Supplementary Figure S2: Representative flow cytometric profiles showing cell cycle parameters; Supplementary Figure S3: Fluorescence microscopy of DU145 prostate cancer cells treated with plagiochiline A or vehicle control; Supplementary Figure S4: Fluorescence microscopy of DU145 prostate cancer cells treated with plagiochiline A or controls, showing cells proceeding through mitosis; Supplementary Figure S5: Plagiochiline A induces cell death in DU145 prostate cancer cells.

## References

[1] Aponte J.C., Vaisberg A.J., Rojas R., Sauvain M., Lewis W.H., Lamas G., Sarasara C., Gilman R.H., Hammond G.B. (2009). A multipronged approach to the study of peruvian ethnomedicinal plants: A legacy of the ICBG-Peru Project. J. Nat. Prod..

[2] Aponte J.C., Yang H., Vaisberg A.J., Castillo D., Malaga E., Verastegui M., Casson L.K., Stivers N., Bates P.J., Rojas R. (2010). Cytotoxic and anti-infective sesquiterpenes present in Plagiochila disticha (Plagiochilaceae) and Ambrosia peruviana (Asteraceae). Planta Med..

[3] Asakawa Y., Yoyota M., Takemoto T., Kubo I., Nakanishi K. (1980). Insect antifeedant secoaromadendrane-type sesquiterpenes from *Plagiochila* species. Phytochemistry.

[4] Toyota M., Tanimura K., Asakawa Y. (1998). Cytotoxic 2,3-secoaromadendrane-type sesquiterpenoids from the liverwort Plagiochila ovalifolia. Planta Med..

[5] Durán-Peña M.J., Ares J.M.B., Hanson J.R., Collado I.G., Hernández-Galán R. (2015). Biological activity of natural Sesquiterpenoids containing a gem-Dimethylcyclopropane unit. Nat. Prod. Rep..

[6] Jones E.W., Rose F. (1975). *Plagiochila atlantica* F. Rose, sp. nov.—*P. ambagiosa* auct. J. Bryol..

[7] Ramírez M., Kamiya N., Popich S., Asakawa Y., Bardón A. (2010). Insecticidal constituents from the Argentine Liverwort Plagiochila bursata. Chem. Biodivers..

[8] Nagle A., Hur W., Gray N.S. (2006). Antimitotic agents of natural origin. Curr. Drug Targets.

[9] Barr F.A., Gruneberg U. (2007). Cytokinesis: Placing and making the final cut. Cell.

[10] Frenette P., Haines E., Loloyan M., Kinal M., Pakarian P., Piekny A. (2012). An anillin-Ect2 complex stabilizes central spindle microtubules at the cortex during cytokinesis. PLoS ONE.

[11] Fededa J.P., Gerlich D.W. (2012). Molecular control of animal cell cytokinesis. Nat. Cell Biol..

[12] Gulluni F., Martini M., Hirsch E. (2017). Cytokinetic Abscission: Phosphoinositides and ESCRTs Direct the Final Cut. J. Cell. Biochem..

[13] Stoten C.L., Carlton J.G. (2017). ESCRT-dependent control of membrane remodelling during cell division. Seminars in Cell & Developmental Biology.

[14] Thoresen S.B., Campsteijn C., Vietri M., Schink K.O., Liestol K., Andersen J.S., Raiborg C., Stenmark H. (2014). ANCHR mediates Aurora-B-dependent abscission checkpoint control through retention of VPS4. Nat. Cell Biol..

[15] Nähse V., Christ L., Stenmark H., Campsteijn C. (2017). The abscission checkpoint: Making it to the final cut. Trends Cell Biol..

[16] Cheffings T.H., Burroughs N.J., Balasubramanian M.K. (2016). Actomyosin ring formation and tension generation in eukaryotic cytokinesis. Curr. Biol..

[17] Chen A., Arora P.D., McCulloch C.A., Wilde A. (2017). Cytokinesis requires localized β-actin filament production by an actin isoform specific nucleator. Nat. Commun..

[18] Mitsushima M., Aoki K., Ebisuya M., Matsumura S., Yamamoto T., Matsuda M., Toyoshima F., Nishida E. (2010). Revolving movement of a dynamic cluster of actin filaments during mitosis. J. Cell Biol..

[19] Moulding D.A., Blundell M.P., Spiller D.G., White M.R., Cory G.O., Calle Y., Kempski H., Sinclair J., Ancliff P.J., Kinnon C. (2007). Unregulated actin polymerization by WASp causes defects of mitosis and cytokinesis in X-linked neutropenia. J. Exp. Med..

[20] Cragg G.M., Newman D.J. (2005). Plants as a source of anti-cancer agents. J. Ethnopharmacol..

[21] Ngan V.K., Bellman K., Hill B.T., Wilson L., Jordan M.A. (2001). Mechanism of mitotic block and inhibition of cell proliferation by the semisynthetic Vinca alkaloids vinorelbine and its newer derivative vinflunine. Mol. Pharmacol..

[22] Jordan M.A., Wilson L. (1998). Microtubules and actin filaments: Dynamic targets for cancer chemotherapy. Curr. Opin. Cell Biol..

[23] Atilla-Gokcumen G.E., Castoreno A.B., Sasse S., Eggert U.S. (2010). Making the cut: The chemical biology of cytokinesis. ACS Chem. Biol..

[24] Matsuo Y., Park J.H., Miyamoto T., Yamamoto S., Hisada S., Alachkar H., Nakamura Y. (2014). TOPK inhibitor induces complete tumor regression in xenograft models of human cancer through inhibition of cytokinesis. Sci. Transl. Med..

[25] Chircop M., Perera S., Mariana A., Lau H., Ma M.P., Gilbert J., Jones N.C., Gordon C.P., Young K.A., Morokoff A. (2011). Inhibition of dynamin by dynole 34-2 induces cell death following cytokinesis failure in cancer cells. Mol. Cancer Ther..

[26] Joshi S., Perera S., Gilbert J., Smith C.M., Mariana A., Gordon C.P., Sakoff J.A., McCluskey A., Robinson P.J., Braithwaite A.W. (2010). The dynamin inhibitors MiTMAB and OcTMAB induce cytokinesis failure and inhibit cell proliferation in human cancer cells. Mol. Cancer Ther..

[27] Greenbaum M.P., Iwamori T., Buchold G.M., Matzuk M.M. (2011). Germ cell intercellular bridges. Cold Spring Harb. Perspect. Biol..

[28] Lordier L., Jalil A., Aurade F., Larbret F., Larghero J., Debili N., Vainchenker W., Chang Y. (2008). Megakaryocyte endomitosis is a failure of late cytokinesis related to defects in the contractile ring and Rho/Rock signaling. Blood.

[29] Fujiwara T., Bandi M., Nitta M., Ivanova E.V., Bronson R.T., Pellman D. (2005). Cytokinesis failure generating tetraploids promotes tumorigenesis in p53-null cells. Nature.

[30] Cortez B.A., Teixeira P.R., Redick S., Doxsey S., Machado-Santelli G.M. (2016). Multipolar mitosis and aneuploidy after chrysotile treatment: A consequence of abscission failure and cytokinesis regression. Oncotarget.

[31] Lin C.C., Chang M.C., Chang H.H., Wang T.M., Tseng W.Y., Tai T.F., Yeh H.W., Yang T.T., Hahn L.J., Jeng J.H. (2009). Areca nut-induced micronuclei and cytokinesis failure in Chinese hamster ovary cells is related to reactive oxygen species production and actin filament deregulation. Environ. Mol. Mutagen..

[32] Agromayor M., Martin-Serrano J. (2013). Knowing when to cut and run: Mechanisms that control cytokinetic abscission. Trends Cell Biol..

[33] Carmena M. (2012). Abscission checkpoint control: Stuck in the middle with Aurora B. Open Biol..

[34] Chen C.T., Hehnly H., Doxsey S.J. (2012). Orchestrating vesicle transport, ESCRTs and kinase surveillance during abscission. Nat. Rev. Mol. Cell Biol..

[35] Mierzwa B., Gerlich D.W. (2014). Cytokinetic abscission: Molecular mechanisms and temporal control. Dev. Cell.

[36] Abreu P.A., Sousa T.S., Jimenez P.C., Wilke D.V., Rocha D.D., Freitas H.P., Pessoa O.D., La Clair J.J., Costa-Lotufo L.V. (2014). Identification of pyrroloformamide as a cytokinesis modulator. ChemBioChem.

[37] Atilla-Gokcumen G.E., Bedigian A.V., Sasse S., Eggert U.S. (2011). Inhibition of glycosphingolipid biosynthesis induces cytokinesis failure. J. Am. Chem. Soc..

[38] Bai R., Verdier-Pinard P., Gangwar S., Stessman C.C., McClure K.J., Sausville E.A., Pettit G.R., Bates R.B., Hamel E. (2001). Dolastatin 11, a marine depsipeptide, arrests cells at cytokinesis and induces hyperpolymerization of purified actin. Mol. Pharmacol..

[39] Castoreno A.B., Smurnyy Y., Torres A.D., Vokes M.S., Jones T.R., Carpenter A.E., Eggert U.S. (2010). Small molecules discovered in a pathway screen target the Rho pathway in cytokinesis. Nat. Chem. Biol..

[40] Clark A.G., Sider J.R., Verbrugghe K., Fenteany G., von Dassow G., Bement W.M. (2012). Identification of small molecule inhibitors of cytokinesis and single cell wound repair. Cytoskeleton (Hoboken).

[41] Grace K.J., Medina M., Jacobs R.S., Wilson L. (1992). Selective inhibition of cytokinesis in sea urchin embryos by the marine natural product pseudopterolide. Mol. Pharmacol..

[42] Matesic D.F., Villio K.N., Folse S.L., Garcia E.L., Cutler S.J., Cutler H.G. (2006). Inhibition of cytokinesis and akt phosphorylation by chaetoglobosin K in ras-transformed epithelial cells. Cancer Chemother. Pharmacol..

[43] Nakayama Y., Saito Y., Soeda S., Iwamoto E., Ogawa S., Yamagishi N., Kuga T., Yamaguchi N. (2014). Genistein induces cytokinesis failure through RhoA delocalization and anaphase chromosome bridging. J. Cell. Biochem..

[44] O’Brien E.T., Asai D.J., Jacobs R.S., Wilson L. (1989). Selective inhibition of cytokinesis in sea urchin embryos by low concentrations of stypoldione, a marine natural product that reacts with sulfhydryl groups. Mol. Pharmacol..

[45] Zullo K.M., Guo Y., Cooke L., Jirau-Serrano X., Mangone M., Scotto L., Amengual J.E., Mao Y., Nandakumar R., Cremers S. (2015). Aurora A Kinase Inhibition Selectively Synergizes with Histone Deacetylase Inhibitor through Cytokinesis Failure in T-cell Lymphoma. Clin. Cancer Res..

