# Supplementary material for: Plagiochiline A Inhibits Cytokinetic Abscission and Induces Cell Death
Source: Molecules. 2018 Jun 12;23(6):1418. doi: 10.3390/molecules23061418 (PMC6099941; doi:10.3390/molecules23061418)
Supplement: Supplementary file 1 [file molecules-23-01418-s001.pdf]

Supplementary Figure S1

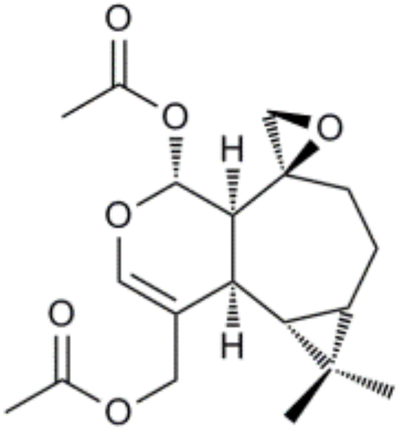

1

Supplementary Figure S1. *Structure of plagiochiline A.*

Supplementary Figure S2

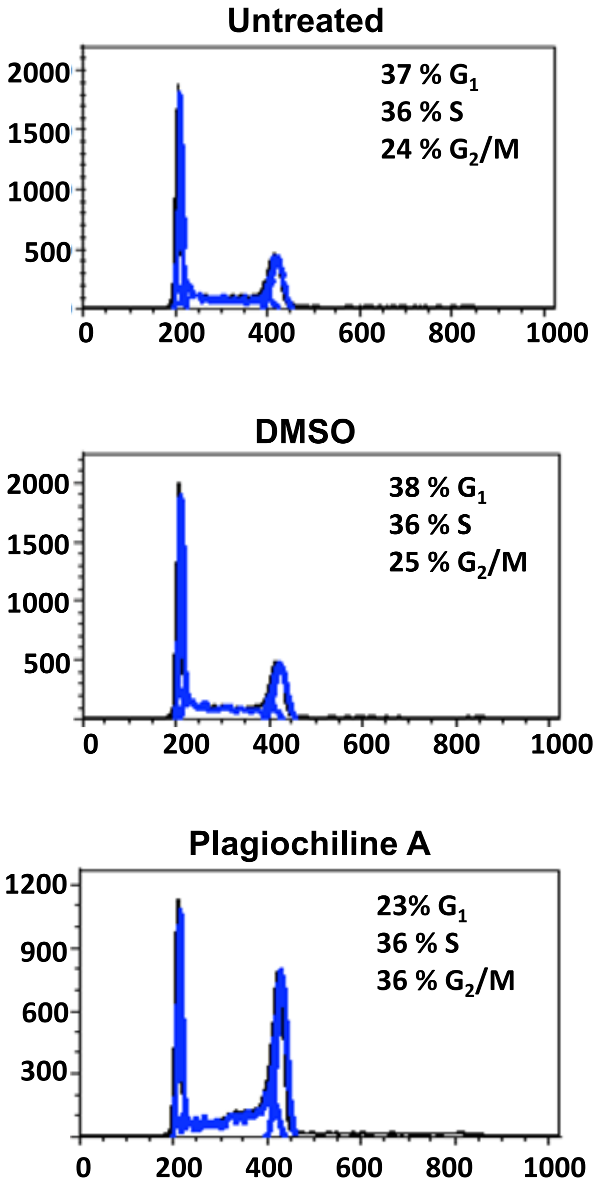

Supplementary Figure S2. *Representative flow cytometric profiles showing cell cycle parameters.* DU145 cells were plated 24 h at 37°C before treatment. Cells were treated with 5 µM plagiochiline A or vehicle control (DMSO) or left untreated for 24 h. After treatment, cells were harvested, fixed and stained with propidium iodide. Cells were analyzed on a FACScalibur flow cytometer. Each histogram indicates the percent of cells in G_1, S, and G_2/M phases of the cell cycle. Data were gated to exclude debris and apoptotic (sub-G1) cells for these calculations.

Supplementary Figure S3

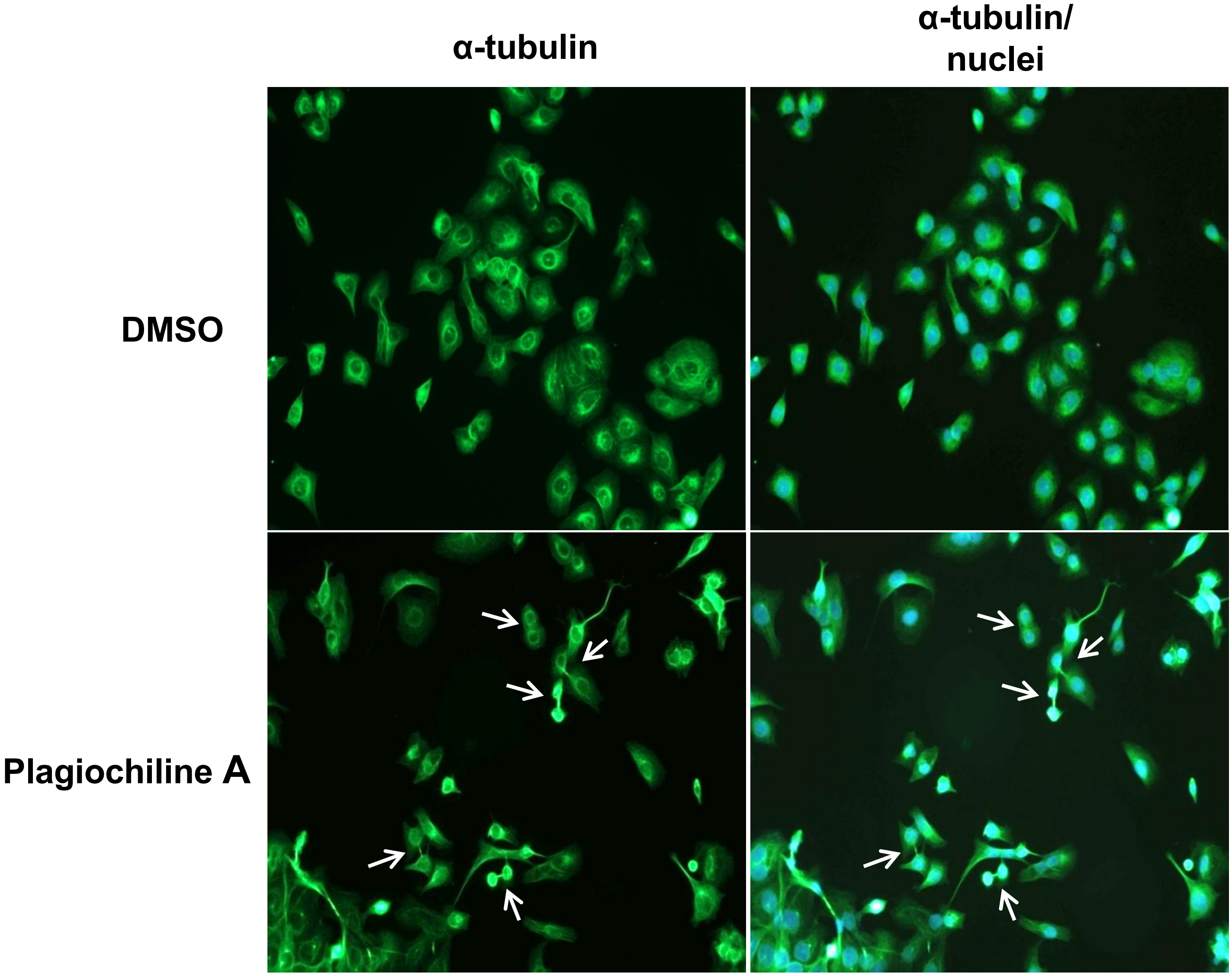

Supplementary Figure S3. *Fluorescence microscopy of DU145 prostate cancer cells treated with plagiochiline A or vehicle control.* DU145 cells were plated on 18 mm diameter glass cover slips and incubated 24 hours at 37°C. Cells were treated with 5 μM plagiochiline A or vehicle control (DMSO) for 48 hours. After treatment, cells were washed with PBS, fixed with 4% paraformaldehyde, and permeabilized. Nuclei were stained with 4'6-diamidino-2-phenylindole (DAPI, blue). α-Tubulin was stained with anti-α-tubulin antibody labeled with fluorescein isothiocyanate (FITC, green). Arrows represent cells in late cytokinesis.

Supplementary Figure S4

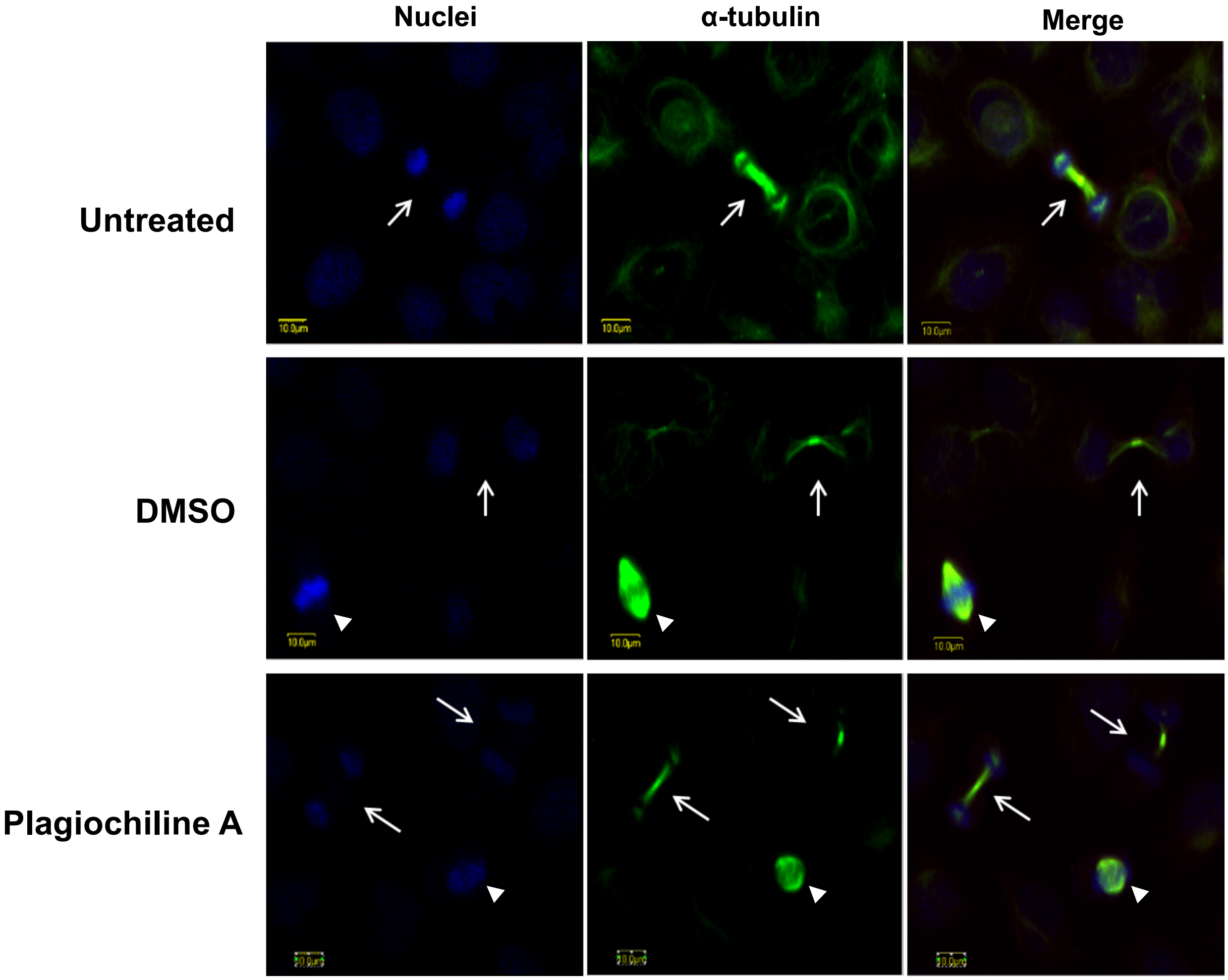

Supplementary Figure S4. *Fluorescence microscopy of DU145 prostate cancer cells treated with plagiochiline A or controls, showing cells proceeding through mitosis.* DU145 cells were plated on 18 mm diameter glass cover slips and incubated 24 h at 37°C. Cells were untreated or treated with 5 μM plagiochiline A or vehicle control (DMSO) for 48 h. After treatment, cells were washed with PBS, fixed with 4% paraformaldehyde, and permeabilized with 1% Triton X-100. Nuclei were stained with 4'6-diamidino-2-phenylindole (DAPI, blue). α-Tubulin was stained with anti-α-tubulin antibody labeled with fluorescein isothiocyanate (FITC, green). Note that the morphology of cells in late cytokinesis (arrows) is similar between plagiochiline-treated and control samples, and that cells at earlier stages of mitosis (arrowheads) are visible. These results suggest that mitosis proceeds normally in plagiochiline A-treated cells up to the point of abscission.

Supplementary Figure S5

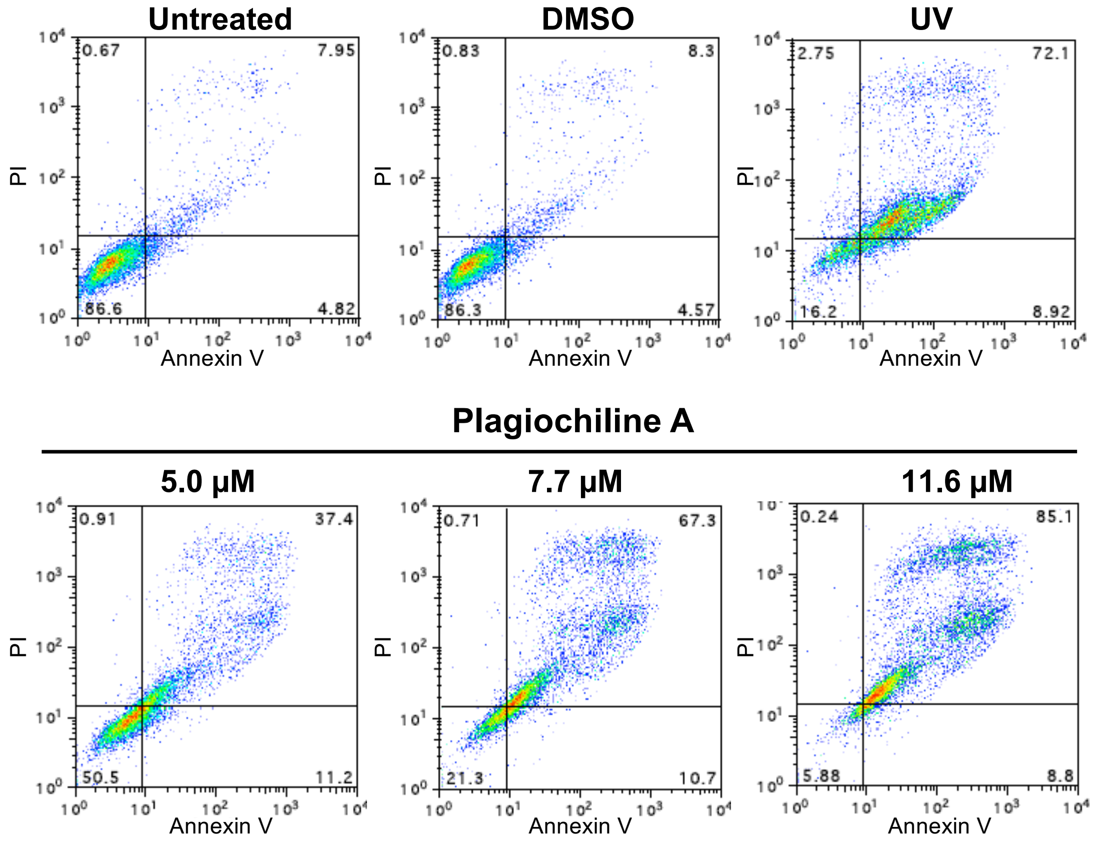

Supplementary Figure S5. *Plagiochiline A induces cell death in DU145 prostate cancer cells***.** DU145 cells were plated 24 h at 37°C before treatment. Cells were treated for 24 h with various concentrations of plagiochiline A, an equivalent volume of vehicle (DMSO), or were left untreated. Some cells were irradiated with ultraviolet light (UV) as a positive control. Cells were subsequently harvested by trypsinization, washed with PBS, and incubated with Annexin V conjugated to FITC and the with propidium iodide (PI). Cells were analyzed for Annexin V and PI staining on a FACScalibur flow cytometer. Each histogram was split in four quadrants to indicate the percent of viable cells (lower left quadrant), early apoptotic cells (lower right quadrant), and late apoptotic or necrotic cells (upper right quadrant) based on their relative PI and Annexin V staining. (The small number of PI-positive cells in the upper left quadrant are presumably non-viable cells that failed to stain strongly for Annexin V).
